# Primary Hyperaldosteronism in End-Stage Renal Disease: Diagnostic Challenges and Treatment Considerations

**DOI:** 10.7759/cureus.9599

**Published:** 2020-08-07

**Authors:** Raj Chand, Srijan Tandukar, Sadiyah Asmil, Michelle Chico

**Affiliations:** 1 Endocrinology and Diabetes, Center for Endocrinology and Diabetes, Shreveport, USA; 2 Transplant Nephrology, John C. Mcdonald Regional Transplant Center – Willis Knighton Health System, Shreveport, USA; 3 Internal Medicine, Melaka Manipal Medical College, Melaka, MYS

**Keywords:** primary hyperaldosteronism, adrenal incidentaloma, aldosterone renin ratio, adrenal adenoma, end-stage renal disease, aldosterone-producing adenoma

## Abstract

An adrenal incidentaloma is defined as an adrenal mass measuring at least 1 cm that is discovered surreptitiously in an imaging study done for reasons other than the evaluation of adrenal disease. The increase in the prevalence of adrenal incidentalomas has paralleled the increase in diagnostic imaging done for evaluation of other abdominal pathologies. However, most of these adrenal incidentalomas are benign non-hyperfunctioning adenomas. When an adrenal incidentaloma is discovered, the simultaneous presence of hypokalemia, metabolic alkalosis, mild hypernatremia, and mild to severe drug-resistant hypertension may alert a clinician to underlying primary hyperaldosteronism. We present a case of adrenal incidentaloma noted in a patient with end-stage renal disease on hemodialysis which presented a diagnostic challenge due to the correction of metabolic parameters with hemodialysis. The patient was found to have an aldosterone-producing adenoma based on an elevated aldosterone-to-renin ratio and was started on a mineralocorticoid antagonist.

## Introduction

Primary hyperaldosteronism (PA) is suspected when a patient presents with hypokalemia, metabolic alkalosis, mild hypernatremia, and mild to severe hypertension [1]. However, the diagnosis of PA presents a diagnostic challenge in end-stage renal disease (ESRD) patients due to the correction of these laboratory parameters with maintenance hemodialysis [2]. We describe a case of adrenal incidentaloma that was later diagnosed as PA secondary to adrenal adenoma in a hemodialysis-dependent ESRD patient.

## Case presentation

**Figure 1:**
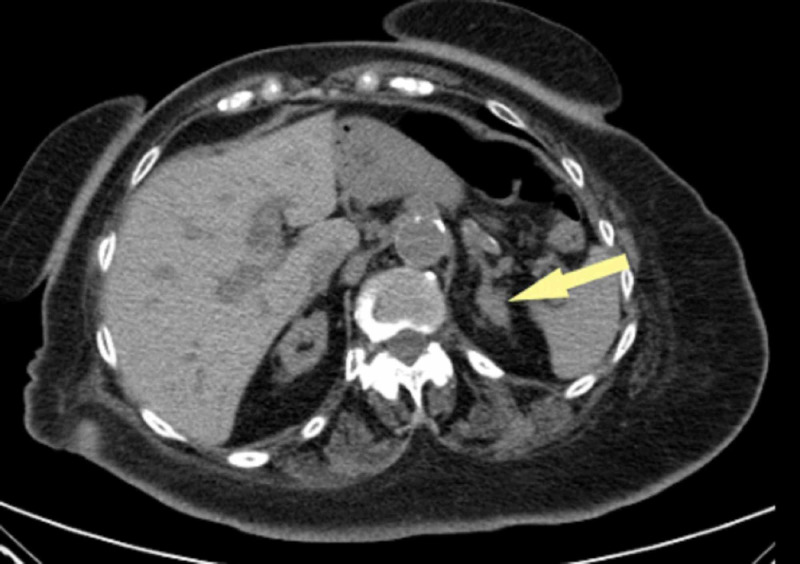
CT scan of the abdomen and pelvis without contrast showing a low-attenuation mass in the left adrenal gland measuring 15.8 mm. An atrophic kidney is seen on the right side.

**Figure 2:**
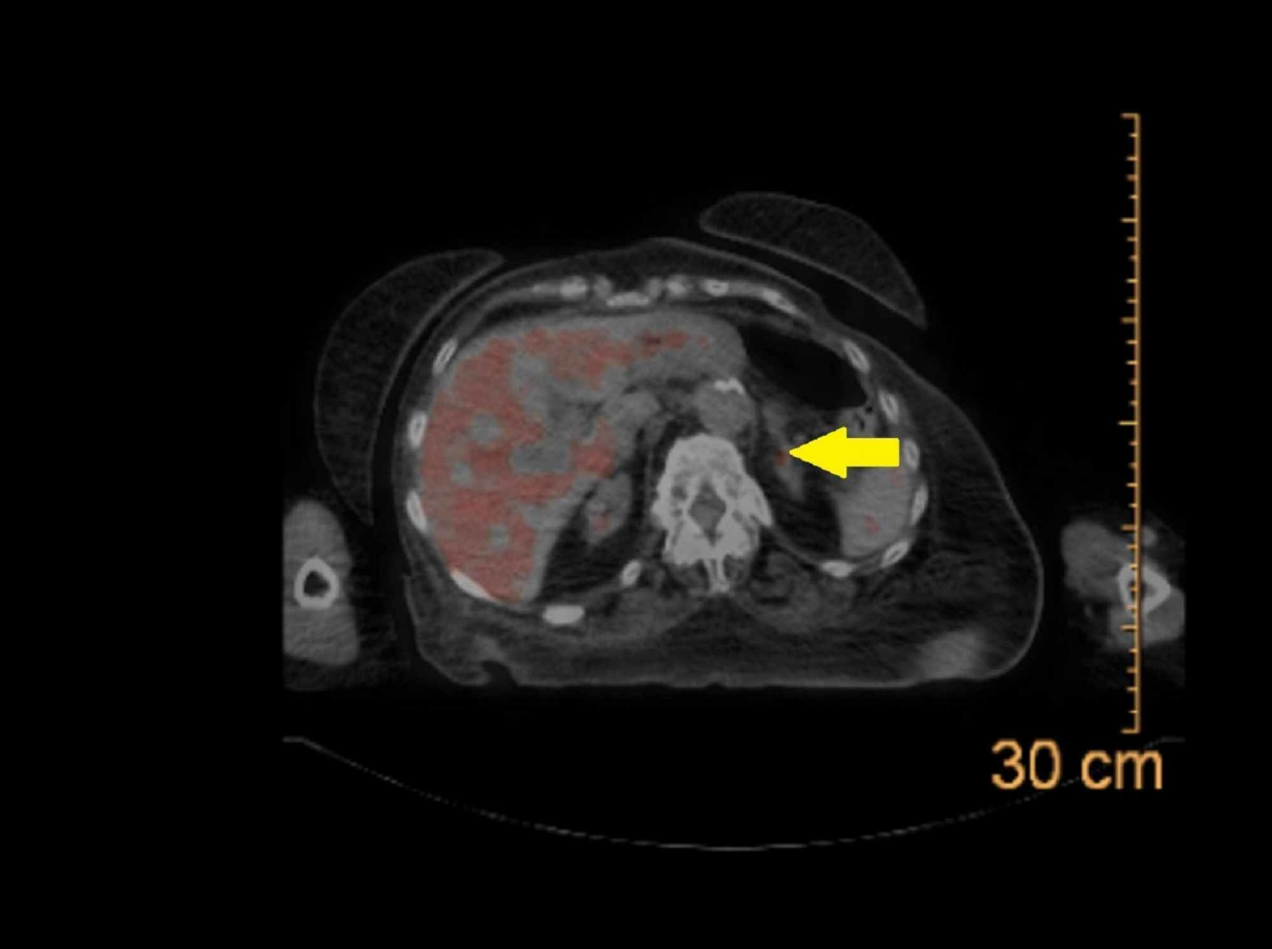
After administering FDG infusion, PET-CT scan demonstrated a metabolically active prominent left adrenal gland with a maximum SUV of 4 and a volume of 1.6 cc. FDG, 18F-fluorodeoxyglucose; PET, positron emission tomography; SUV, standardized uptake value.

A 71-year-old African American female with a history of ESRD on maintenance hemodialysis secondary to type 2 diabetes mellitus was found to have a lung nodule on chest CT scan. She denied any cough, chest pain, fever, fatigue, episodic palpitations with headaches, hirsutism, and weight gain or loss. Her blood pressures predialysis ranged from 96/60 to 112/62 mmHg, without the need for any antihypertensive medications. Her home medications included sevelamer 800 mg twice a day, cinacalcet 30 mg daily, and levothyroxine 50 mcg daily. Her lab chemistries were within acceptable limits as she was on maintenance hemodialysis. She was subsequently evaluated with a CT scan of the abdomen and pelvis that showed a 1.5-cm left adrenal mass (Figure 1). A positron emission tomography CT (PET-CT) scan showed a metabolically active prominent left adrenal gland with a maximum standardized uptake value (SUV) of 4 and a volume of 1.6 cc (Figure 2). Hormonal studies of the adrenal gland showed a high plasma aldosterone level of 210 ng/dL (reference value ≤21 ng/dL) and low plasma renin activity (PRA) of <0.2 ng/ml/hr, with an aldosterone-to-renin ratio (ARR) of 1,050. Measurement of 7 AM cortisol level and fractionated plasma metanephrine was 16.5 ug/dL (reference range 4.5-22.7 ug/dL) and 0.2 nmol/L (reference value <0.50 nmol/L), respectively. Urinary 24-hour cortisol, catecholamine, and metanephrine levels could not be performed as the patient was anuric. The patient was started on spironolactone as she was not a surgical candidate.

## Discussion

An adrenal mass greater than 1 cm in diameter that is discovered incidentally by a radiologic examination done for other reasons is known as an adrenal incidentaloma [3]. Although most adrenal incidentalomas are clinically benign non-hyperfunctioning adrenocortical adenomas, a workup for malignancy and a hyperfunctioning gland is recommended [3,4]. The incidence of adrenal incidentaloma is around 4% and the prevalence of PA in patients with adrenal incidentaloma is relatively low, ranging from 1.5% to 7% [5,6]. This is probably due to the definition of adrenal incidentaloma that excludes patients with hypertension and hypokalemia. We describe a rare case of aldosterone-producing incidentaloma in a patient with ESRD on hemodialysis.

PA classically presents with hypertension, hypokalemia, and metabolic alkalosis. The mechanism of hypokalemia is mediated through the accelerated renal excretion of potassium. Hypertension is due to the increased sodium reabsorption in the renal collecting tubules and the consequent hypervolemia. However, resistant hypertension may be the only sign for primary hyperaldosteronism in patients with ESRD receiving hemodialysis. The absence of hypokalemia is attributed to the decreased ability of the kidneys to excrete potassium in patients with ESRD due to concomitant anuria. Due to maintenance hemodialysis, our patient did not have any imbalance in electrolytes or acid-base status.

A large study suggests how quantitative plasma ARR can dramatically improve the diagnostic accuracy of PA. The ARR of more than 100 has a specificity close to 100% for the diagnosis of aldosterone-producing adenoma (APA) [7]. Our patient was found to have an adrenal incidentaloma on a CT scan (Figure 1). Further evaluation revealed an elevated plasma aldosterone concentration (PAC) with low PRA. The ARR was found to be 350, highly suggestive of an APA.

The diagnosis of PA in patients with ESRD can be challenging. After initial screening with PAC and PRA levels, the confirmatory tests such as 24-hour urine aldosterone or oral sodium loading, cannot be routinely performed due to anuria. A previous case report of resistant hypertension in a patient with ESRD utilized low-dose spironolactone to evaluate renin response. With the normalization of blood pressure and renin levels, the diagnosis of PA was confirmed [8]. For our patient, the diagnosis of APA was supported by performing a PET-CT scan. The results are shown in Figure 2. Other confirmatory studies, such as oral sodium loading and adrenal vein sampling test, were deferred due to anuria and being a non-surgical candidate, respectively.

Recent research suggests that the actions of aldosterone are beyond its renal and hemodynamic effects [9,10]. Aldosterone has physiological and pathophysiological effects in non-epithelial tissues, including the heart, vasculature, and brain. These effects are independent of its action on renal tubular cell ion transport. A large retrospective study shows an increased rate of cardiovascular events in patients with primary aldosteronism. These include stroke, myocardial infarction, and atrial fibrillation, which were seen independent of blood pressure changes [7]. Even though our patient was normotensive, we decided to place the patient on a mineralocorticoid antagonist for long-term cardiovascular benefits. It is important to note the substantial evidence associating an increased aldosterone level with glomerular hyperfiltration and the accelerated progression of chronic kidney disease [10]. Primary aldosteronism in our patient could be a hypothetical cause for chronic kidney disease and ESRD.

## Conclusions

Currently, there are no specific guidelines for the management of PA in patients with ESRD. We suggest the use of a quantitative ARR for the diagnosis and confirmation in these patients. Further lateralization of PA can be done using a high-resolution CT scan with fine cuts. Non-surgical candidates with APA should be primarily treated with a mineralocorticoid antagonist.
